# A guide to analysis and reconstruction of serial block face scanning electron microscopy data

**DOI:** 10.1111/jmi.12676

**Published:** 2018-01-15

**Authors:** E. COCKS, M. TAGGART, F.C. RIND, K. WHITE

**Affiliations:** ^1^ Institute of Genetic Medicine Newcastle University Central Parkway Newcastle upon Tyne NE1 3BZ UK; ^2^ Institute of Neuroscience Newcastle University Framlington Place Newcastle upon Tyne NE2 4HH UK; ^3^ Electron Microscopy Research Services Newcastle University Medical School Framlington Place Newcastle upon Tyne NE2 4HH UK

**Keywords:** Amira, blender, Fiji, image analysis, microscopy image browser, serial block face scanning electron microscopy, skeletal muscle

## Abstract

Serial block face scanning electron microscopy (SBF‐SEM) is a relatively new technique that allows the acquisition of serially sectioned, imaged and digitally aligned ultrastructural data. There is a wealth of information that can be obtained from the resulting image stacks but this presents a new challenge for researchers – how to computationally analyse and make best use of the large datasets produced. One approach is to reconstruct structures and features of interest in 3D. However, the software programmes can appear overwhelming, time‐consuming and not intuitive for those new to image analysis. There are a limited number of published articles that provide sufficient detail on how to do this type of reconstruction. Therefore, the aim of this paper is to provide a detailed step‐by‐step protocol, accompanied by tutorial videos, for several types of analysis programmes that can be used on raw SBF‐SEM data, although there are more options available than can be covered here. To showcase the programmes, datasets of skeletal muscle from foetal and adult guinea pigs are initially used with procedures subsequently applied to guinea pig cardiac tissue and locust brain. The tissue is processed using the heavy metal protocol developed specifically for SBF‐SEM. Trimmed resin blocks are placed into a Zeiss Sigma SEM incorporating the Gatan 3View and the resulting image stacks are analysed in three different programmes, Fiji, Amira and MIB, using a range of tools available for segmentation. The results from the image analysis comparison show that the analysis tools are often more suited to a particular type of structure. For example, larger structures, such as nuclei and cells, can be segmented using interpolation, which speeds up analysis; single contrast structures, such as the nucleolus, can be segmented using the contrast‐based thresholding tools. Knowing the nature of the tissue and its specific structures (complexity, contrast, if there are distinct membranes, size) will help to determine the best method for reconstruction and thus maximize informative output from valuable tissue.

## Lay Abstract

Electron microscopes have been used for decades to image cellular detail at high magnification. However the resulting images are 2‐dimensional. The development of an electron microscope in which we can section tissue in situ means that we can now acquire stacks of images giving us detail in 3‐dimensions. However the challenge with these large datasets is to reconstruct features of interest to form a 3D model. There are a number of computer programs that can be used to do this but they are not intuitive and can be overwhelming for researchers new to them. Here we explain terminology used in the programs and provide a step‐by‐step guide with accompanying videos to help researchers get started with their image analysis and get the best from their data.

## Introduction

Electron microscopy (EM) has evolved to incorporate different preparation techniques for the imaging of a wide variety of samples. Transmission and scanning EM (TEM and SEM, respectively) are regularly used to analyse biological material in order to reveal structural information, which may relate to function. Yet, there are limitations. For example, SEM generally images the surface topography of cells and tissues but not intracellular structures (unless freeze‐fracture techniques are used). On the other hand, TEM provides information on spatial arrangements within cells to within 1 nm resolution. However, this is accomplished by examining a single ultrathin section, typically 50–100 nm, from a much larger sample, which could range from ∼10 μm (single cells) to several millimetres (tissues). Therefore, important information regarding spatial arrangements of structures of interest through the depth of a cell or tissue is difficult to obtain. Manual serial‐sectioning can be combined with TEM to create 3D stacks and visualizations of data (Andersson‐Cedergren, 1959; Kristen & Stevens, 1988; Bock *et al*., 2011; Takemura, 2015; Lee *et al*., 2016). However, this is extremely time‐consuming, requires a high level of experience in microtomy and manual image alignment and, even then, often results in damaged or lost sections whose ‘missing’ information has to be interpolated.

The desire within the EM community to obtain data in three spatial dimensions (*x*–*y*–*z*) at the ultrastructural level led to the development of a rudimentary automated system for serial sectioning coupled with EM imaging by Kuzirian and Leighton (1983). Although it was another two decades before the procedure known as serial block face SEM (SBF‐SEM) was described in published form by (Denk & Horstmann, 2004). It consists of a mini‐ultramicrotome with diamond knife inside an SEM chamber. The knife cuts ultrathin sections from a piece of tissue embedded in a resin block, an electron beam scans the block surface and a detector records the backscattered electrons, producing a digital image. This process is repeated at an operator‐specified depth to produce a digitized stack of aligned images. It is possible to obtain tens to hundreds of serial sections from resin blocks, and aligned images, in a few hours. In addition to SBF‐SEM, there are other methods of 3D volume collection that have been developed, such as array tomography and focused ion beam scanning EM (FIB‐SEM) (reviewed by Peddie & Collinson, 2014; Titze & Genoud, 2016). All techniques produce large quantities of data, which are difficult to manage and time‐consuming to analyse. In this manuscript, we concentrate on data obtained by SBF‐SEM but the same analyses we describe may be applied to datasets obtained by other methods.

The stack of images obtained from biological samples allows researchers to follow cell‐to‐cell arrangements, or intracellular structures, in the *z*‐axis in a number of ways. First, this can be achieved by simply scrolling through the images for qualitative assessment of the features of interest. Second, image analysis software can be used to create 3D reconstructions of the data. These can aid the qualitative assessment of the data, for example, creating movies that show the reconstructions on rotating axis (Kasthuri *et al*., 2015). Third, such tools can also be used for detailed quantification of the biological data. The image analysis can be the most complex and time‐consuming portion of the whole process.

In many ways, the challenges of 3D EM have now shifted from how to capture the difficult‐to‐measure to what to do with all this data? At the outset of an experiment one, ideally, it needs to know how the resultant images are to be analysed. These considerations vary from simple to complex, depending upon the experimental question, the tissue or cell constituency, the resolutions of structures of interest and their contrast to neighbouring structures.

The most likely requirement is the creation of 3D reconstructions of the image stacks, whether for qualitative or quantitative assessment. There are a number of image analysis packages that assist with creating reconstructions (for a recent review of these, see Borrett and Hughes (2016)), each requiring segmentation as a first step. Segmentation is the process of annotating a specific structure on each image so as to follow it in each consecutive image in the *z*‐axis. There are two main ways of accomplishing this. In one case, the object(s) is highlighted manually by adding a coloured layer(s) on top of the image. An alternative process involves assigning individual image pixels to only one object, which means if a pixel is reselected during another segmentation, it will be reassigned.

The methods of segmentation can be further divided into manual, semiautomated and automated categories. Manual segmentation tools require the user to annotate the object, e.g. by colouring, over every slice. Semiautomated tools use a combination of user input and programme predictions, to highlight a structure. An example of this is interpolation. The user manually annotates the structure every *n*th slice and the programme will fill in the empty slices using the annotated image as a guide to predict the possible shape of the object. Another is the thresholding tool, which selects pixels based on the contrast limits set by the user. These limits allow for the selection of light or dark pixels depending on the appearance of the object. There is the option available in some programmes for machine learning automated segmentation. Here, the programme ‘learns’ object selection based on trial runs performed by the user on a sample dataset. These settings are then automatically applied to the full dataset to be analysed and can be implemented on other datasets as well.

The complexity of SBF‐SEM datasets, and scenario‐specific analysis requirements, can make it difficult to know which image analysis tools and procedures to follow to make best interpretations of the data. Therefore, the purpose of this paper is to compare three popular image analysis programmes, and the segmentation tools they offer, for the qualitative and quantitative analysis of SBF‐SEM data. We provide detailed protocols for the data handling and analyses. In doing so, we aim to provide direction to researchers new to SBF‐SEM by drawing attention to advantages and limitations of the software packages and tools. A similar comparison was done by Tsai *et al*. (2014), which details data reconstruction from multiple volume EM techniques. However, the in‐depth workflow from these techniques may appear daunting for those new to image analyses. This paper is aimed at researchers with no or little experience using image analysis software and SBF‐SEM and provides a simple workflow for deciding which methods to use.

The material analysed is predominantly guinea pig skeletal muscle, chosen because it exhibits regular, well‐defined intracellular structures by EM that allowed us to test the effectiveness of each software tool. From the results, we developed a decision‐making map that can be applied to the analysis of any structure from any sample type and will help researchers choose the workflow to apply to their SBF‐SEM data. By following this workflow, the researcher will ensure that they are in the best position to address the aims of their study and maximize the output from this relatively new technique.

## Method

### Tissue preparation for SBF‐SEM

Skeletal muscle tissue (psoas and soleus muscles) and hearts, from Duncan Hartley guinea pigs, were terminated under licenced procedures according to the Animals Scientific Procedures Act 1986 (ASPA). They were then microdissected into 2% glutaraldehyde with 0.1M sodium cacodylate buffer and left for a minimum of 12 h in the fixative at 4°C. The samples then undergo a heavy metal staining protocol (Wilke *et al*., 2013). The tissues were washed in 0.1M sodium cacodylate pH7.4 followed by a solution of 3% potassium ferricyanide with 2% aqueous osmium tetroxide in ddH_2_O for 1 h. Then, followed by filtered 10% thiocarbohydrazide, TCH, for 20 min and then secondary 2% osmium tetroxide for 30 min. The samples were then placed in 1% uranyl acetate at 4°C overnight followed by lead aspartate solution, 0.12 g of lead nitrate in 20 mL aspartic acid for 30 min.

The brain from an adult locust (*Locusta migratoria*) was sacrificed by ice, dissected in cold saline and placed in 2% paraformaldehyde, 2.5% glutaraldehyde in 0.1M sodium cacodylate buffer. It was processed with an adapted version of the Wilke *et al*. (2013) protocol described in Wernitznig *et al*. (2016). The main differences to the above protocol are the use of reduced osmium (1%) and a shorter time in uranyl acetate but at 60°C.

Between each step, all samples were washed in several changes of ddH_2_O. The samples were dehydrated with acetone, from 25% to 100% and then impregnated with increasing concentrations of Taab 812 hard resin in acetone with several changes of 100% resin. The samples were embedded into 100% resin and left to polymerize at 60°C for a minimum of 36 h.

The resin blocks were trimmed using a razor blade to form a trapezoid block face. Using a diamond knife, 1 μm sections are taken and stained with toluidine blue and viewed under a light microscope. Several 70 nm sections were taken, placed on a copper grid and viewed on a CM100 TEM (FEI), to check tissue morphology and orientation and penetration of the staining. This is done to ensure that the tissue has been adequately processed for viewing by SBF‐SEM. The blocks were then further trimmed to approximately 0.75 mm × 0.75 mm and glued onto a pin. In order to reduce sample charging within the SEM, the block was painted in silver Dag and sputter‐coated with a 5 nm layer of gold.

### SBF‐SEM settings and image analysis

The specimens were placed into a Zeiss Sigma SEM (Zeiss, Cambridge, UK) incorporating the Gatan 3View (Gatan inc., Abingdon, UK) as the SBF‐SEM system. For this particular project, the following parameters were used. For each sample, the images were obtained at 2.5–5 kV accelerating voltage, with an aperture of 30 μm, in variable pressure ranging from 20 to 53 Pa. The blocks were sectioned (unless stated otherwise) at a thickness of 70 nm and the images recorded at a range of magnifications with a resolution of 1024 × 1024 pixels or 3000 × 3000 pixels with a 20 μs/pixel dwell time and at resolution ranging from 5 to 20 nm.

**Figure 1:**
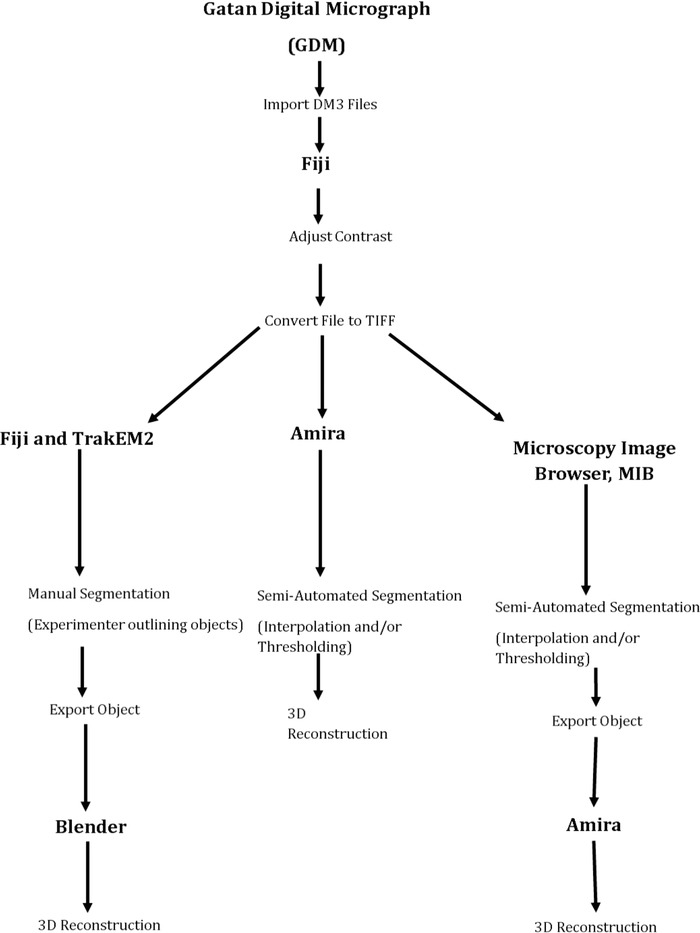
Flow chart showing the steps for the image analysis comparison. The first step is to adjust the contrast and convert the images from DM files formats to TIFF. The stack of TIFFs is then analysed in each of the three programmes, and the examples of the segmentations used in each are also shown. The final step in the process is to reconstruct the segmentations into a 3D model.

Gatan Digital Micrograph was used to collect digitized images of each experimental run in a DM3 format. The data were then analysed using three different image analysis programmes (Fig. 1). These are Fiji (http://fiji.sc/), Amira (http://www.fei.com/software/amira-3d-for-life-sciences/) and Microscopy Image Browser, MIB (http://mib.helsinki.fi/). Fiji, via the plugin TrakEM2 (Cardona *et al*., 2012), is primarily an operator‐driven programme that is freely available. For a comparison of the programmes see Table 1. Amira and MIB (Belevich *et al*., 2016) have the capacity for using semiautomated tools: Amira requires a commercial license and MIB is freely available. Amira enables visualization of all analysed data, whereas FIJI and MIB are purely analytical programmes with basic visualization and require a secondary programme to perform computational analysis of the segmentations. Blender (https://www.blender.org/) is one such programme, it is a free graphics software which can be used to reconstruct the objects created in Fiji with the aid of Neuromoph Tools (Jorstad *et al*., 2015) (http://cvlab.epfl.ch/NeuroMorph) and perform computational analysis. These were developed specifically to import the objects from Fiji and perform quantification analysis. Opening the segmented 3D objects via these tools also ensures that the dimensions are consistent with the parameters from the raw data. In MIB, the segmentation model file can be exported in a variety of formats to different programmes, and in this protocol, Amira is used as the example.

**Table 1:** Comparison of imaging programmes. Examples of the different types of image formats and segmentation tools offered by three analysis programmes

	Fiji	Amira	MIB
Image import format	.tif, .dm3, .png, .bmp	.tif, .png, .bmp	.tif, .dm3, .dm4, .png, .bmp
Object export format	.obj	.am, .surf	.am, .tif, .mat, .model, .stl, .mrc, .mod, .nrrd, .h5
Manual Brush	✓	✓	✓
Interpolation		✓	✓
B/W thresholding		✓	✓
Magic wand thresholding		✓	✓
Watershed/SLIC segmentation			✓

### Protocols for image file handling and analyses

The protocols below are applied initially to the examination of a skeletal muscle SBF‐SEM dataset and the steps in each process illustrated in the accompanying video file tutorials (Supporting files S1–S5). The raw datasets used in the videos can be accessed via the EMPIAR website: http://www.ebi.ac.uk/pdbe/emdb/empiar/ (Accession code: EMPIAR‐10092). The analysis is performed on a Toshiba laptop with Intel® Core i7‐5500U CPU, 2.40 GHz, 16 GB RAM and 64 bit.

### Converting DM3 to TIFF (video Fiji image processing)

The raw data are saved as a DM3 file format from Gatan Digital Micrograph, GDM, which cannot be opened in all imaging analysis programmes. The first step therefore is to convert the images into a TIFF format. During this conversion process, image contrast can be lost and a normalization step is carried out to prevent this.

(1) An image sequence is imported by selecting ‘File’ > ‘Import’ > ‘Image Sequence’ in Fiji *(Supplementary file S1)*.
(2) Select the first image, or entire folder of pertinent images and a new window will appear.
(3) Check the number of images to be imported and ensure that nothing else is ticked.
(4) At this point, the 16 bit images can be converted into 8 bit images, to speed up the processing of the images and reduce the final size of the data.
(5) The image stack will open.
(6) Normalize the contrast by selecting ‘Process’ > ‘Enhance Contrast’.
(7) Change saturated pixels to 1% and select ‘Normalize’ and ‘Process All’.
(8) Contrast can be further enhanced by going to ‘Image’ > ‘Adjust’ >‘Brightness/Contrast’.
(9) Manually adjust the brightness and contrast by moving the sliders.
(10) The image parameters have to be changed by selecting ‘Image’ > ‘Properties’.
(11) The parameters for the *x* and *y* are always correctly registered in Fjji; however, the *z* parameter will need to be changed to the correct thickness of slices taken.
(12) The images are adjusted, if required, using filters (such as denoising) found in ‘Process’ > ‘Noise’ or ‘Process’ > ‘Filters’ for all the other filter options.
(13) Save the adjusted images as TIFF by clicking ‘File’ > ‘Save as Image Sequence’.

The following steps are then undertaken in each programme to segment each of the image stacks:

(1) *Fiji/TrakEM2 (Image Analysis Fiji)*
  (i) The image stack to be analysed is opened in Fiji, as detailed above.
  (ii) To create a new TrakEM2 file, select ‘File’ > ‘New’ > ‘TrakEM2 (Blank)’ *(Supplementary file S2)*.
  (iii) Two new windows will open. One is the object window and the other is the analysis window.
  (iv) Right click and select ‘Import’ >‘Import Stack’ in the analysis window to import the images.
  (v) Right click in the object organizer window and select ‘Anything’ > ‘Add New Child’ > ‘Area_List’.
  (vi) Drag ‘Anything’ into the middle column to create a new folder and then drag ‘Area List’ to create a new object in that folder.
  (vii) Rename the object by right‐clicking and selecting ‘Area_List’ > ‘Rename’.
  (viii) The object will appear under the ‘Z Space’ tab in the analysis window and select it to begin segmentation.
  (ix) Use the brush tool to draw an outline around the selected object on the image.
  (x) Fill the object by holding the shift button and clicking in the centre of the outline.
  (xi) Repeat this over each slice and for each user‐defined object to be segmented.
  (xii) Once the segmentation of a defined object (s) is completed, the results can be viewed in the 3D viewer. Right click in either of the windows and select ‘Show in 3D’.
  (xiii) The 3D reconstruction(s) will appear in a new window.
  (xiv) The model is saved as an .obj file and to be exported to other packages if required.
  (xv) 3D reconstructions created in Fiji can be analysed in Blender.

(2) *Blender v2.76 (Blender)*
  (i) To install the Neuromorph tools, go to ‘Install from File’ > ‘File’ > ‘User Preferences’ *(Supplementary file S3)*.
  (ii) The tools are found in the side panel in the main interface under ‘Misc’.
  (iii) To open the .obj file of interest select the ‘Scene’ tab on the right hand side and then ‘Import Object’.

(3) *Microscopy Image Browser, MIB v2.1 (Image Analysis MIB)*

MIB can be opened through Matlab or as a standalone programme, and the information on how to do either is found on the MIB website (http://mib.helsinki.fi/).

(i) Navigate to the location of the TIFF image stack to be imported by using the ‘Directory Contents’ *(Supplementary file S4)*.
(ii) Highlight all the images to be analysed, right‐click and select ‘Combine Selected Datasets’.
(iii) The image stack will open.
(iv) Change the dataset parameters by going to ‘Dataset’ > ‘Parameters’.
(v) Click ‘Create’, in the ‘Segmentation’ panel and select 63 or 255 models, depending on how many objects will be segmented, to start the segmentation.
(vi) Add a material to the ‘Segmentation’ panel by clicking the ‘plus’ button, change the name of the material and it will appear in the column.
(vii) In MIB, pixels can only be selected and assigned to one material at a time, so segmentation has to be done one at a time.
(viii) Double click on the coloured square to the left of the material name to change the colour.
(ix) A variety of tools can then be used for the segmentation.

**Manual**

(1) Below the material panel is the segmentation tool drop down list. The default when you start the programme will be the ‘Brush’ tool, a manual segmentation tool.
(2) Use the brush tool to trace the outline of the object and repeat this over each slice. Press Shift and F to fill the object throughout all the slices.
(3) Hold the Ctrl button to turn the brush tool into an eraser and remove any errors.
(4) The selected pixels will appear green on the image.
(5) Assign the selection to the ticking the correct material in the ‘Add to’ box and pressing Shift and A.

**Watershed/SLIC**

(1) When the ‘Brush’ tool is selected, there are two more options of segmentation that can be found ‘Watershed’ and ‘SLIC’.
(2) The ‘Watershed’ tool combines the pixels in the image into superpixels, based upon boundaries and changes in contrast.
(3) The size of the superpixels is adjusted by changing the ‘N’ number, the higher the number, the larger the superpixel.
(4) Click on the image using the brush tool and the superpixels appear on the image, as pink outlines, select the superpixels by moving over them using the tool.
(5) The ‘SLIC’ option is a similar tool but the pixels are grouped into superpixels based on similar contrast and instead the smaller the N number, the larger the pixel.
(6) The superpixels will only appear in the area of the image seen in the ‘Image View’ and not over the entire image.

**Interpolation**

(1) Using the brush tool manually, draw on every *n*th slice and then click ‘I’ or go to ‘Selection’ > ‘Interpolation’ and the gaps in the segmentation will be filled in.
(2) Check and correct any errors that have occurred.

  

**Thresholding**

(1) Thresholding is the selection of pixels based on a user inputted contrast range and there are two types, B/W thresholding and the Magic Wand tool.
(2) Select ‘B/W Thresholding’ from the menu. Alter the range by adjusting the two sliders until a correct selection is made over the entire image.
(3) Click ‘All’ for B/W thresholding to be applied to the stack of images.
(4) Chose the ‘MagicWand‐Regiongrowing’ tool, change the variation and radius of the selection tool.
(5) Click on a single pixel in the object, pixels within the range and radius will be selected, alter the variation and radius until the desired selection is made.
(6) Select ‘3D’ in the ‘Selection’ panel to apply the thresholding to the stack.
(7) Check for any errors and correct using the brush tool.

**Saving file**

(1) Once segmentation is finished (by whichever chosen process) go to ‘Model’>’Save Model’ to save the model file in the preferred format.
(2) Recommended file format for the duration of the segmentation the Matlab file format (.model) is preferred and the model will automatically save in this format.

**Saving for opening in Amira**

(1) Save the model file to open in Amira go to ‘Model’>’Save Model As’.
(2) Select any of the ‘.am’ file types.
(3) The new model file can now be opened in Amira (see Reconstruction under Amira).

(4) *Amira v6.0 (Image Analysis Amira)*
  (i) Select ‘Open data’, highlight all the TIFF images and click open to import the images *(Supplementary file S5)*.
  (ii) In the new window change the voxel measurements to the pixel measurements, as mentioned at the beginning.
  (iii) In the main interface, a file will appear which corresponds to the image stack and a single orthoslice will automatically appear in the viewing area on the right.
  (iv) Right click on the main file and select ‘Edit New Label’ to start segmentation and switch to the ‘Segmentation Panel’.
  (v) Click ‘Add’ to add new objects to the segmentation panel, double‐click on it to change the name and right click to change the colour and appearance.
  (vi) The segmentation tools are at the bottom of the right‐hand panel.

**Manual and interpolation**

(1) Select the brush symbol for the manual tool to highlight objects.
(2) Manfully annotate every *n*th slice and select ‘Interpolate’ under the ‘Selection’ tab.
(3) Assign the selection to a label by selecting the ‘plus’ sign, to add selection from all slices select ‘Volume’ and just for the one slice select ‘current slice’.
(4) To remove the selection click the ‘minus’ sign.

  

**Thresholding**

(1) Click on the magic wand symbol to use the magic wand thresholding tool.
(2) A graph will appear with sliders.
(3) Select a pixel on the image and a pointer will appear on the graph to show where on the contrast scale that pixel appears.
(4) An area will be highlighted and manually adjust the sliders to change the pixels selected until the correct selection is made.
(5) Tick ‘3D’ to perform the action across all slices.
(6) To view the object on 3D in the segmentation panel choose the four‐panel viewer above the viewing window.

**Reconstruction**

(1) Select the project panel tab once the segmentation is complete, where there will be a secondary file connected to the original image file with .labels extension (corresponding to the new objects created in the segmentation panel).
(2) Right click the .Labels file and select ‘Generate Surface’ > ‘Apply’.
(3) Another file will appear connected to the .Labels file, a .surf file.
(4) Right click the .surf file and select ‘Surface View’, a render of the 3D model will appear in the viewing window.

## Results

**Figure 2:**
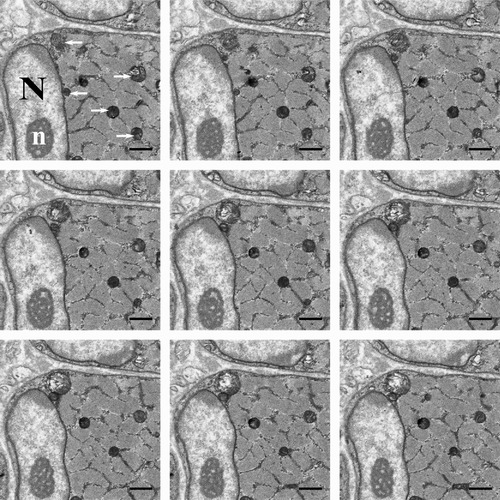
Example of SBF‐SEM image series. Nine consecutive images (viewed from left to right) from a stack of 93 serial images of a portion of a muscle cell from the skeletal muscle psoas from a late foetus guinea pig. In the first image, the nucleus can be seen, labelled with an ‘N’, as well as the nucleolus, white ‘n’, and the mitochondria, labelled with white arrows. Over each slice, of 70 nm, the structures change shape, as shown in the images. The images were taken at 12k× magnification, 7 nm resolution and an image size of 1024 × 1024 pixels. All scale bars are 1 μm.

Figure 2 illustrates a montage of serial‐sectioned raw data SBF‐SEM images from the psoas skeletal muscle (late foetus). Nine consecutive images are shown that are part of a larger dataset of 93 serial images. This dataset was used to illustrate the application of the different image analysis programmes and protocols. From the data, the nucleus, nucleolus, chromatin and mitochondria were all segmented and reconstructed using the tools detailed in the methods. Further information on which tool was specifically used for each structure is included in the following results and figures. The methods of segmentation can be further divided into manual, semiautomated and automated categories. Manual segmentation tools require the user to annotate the object, e.g. by colouring over every slice. Semiautomated tools use a combination of user input and programme predictions to highlight a structure. Automated segmentation requires no user input, although some machine learning is often involved; however, this method is not used in this analysis.

A timing analysis was also performed over 20 slices for each different method used to segment the nuclei and mitochondria (see Table 2). Interpolation was used to segment the nuclei in MIB and Amira, which was quicker than the manual segmentation performed in Fiji, with MIB being the fastest between all three programmes. The thresholding method in Amira was the quickest method to segment the mitochondria. Overall the semiautomated tools, Amira specifically, segmented the structures fastest, when combining the nuclei and mitochondria timing analysis. Note that the speed of the semiautomated procedures will be dependent on computing ability.

**Table 2:** Timing analysis results. The table shows the results from the timing analysis performed on a small portion of the total skeletal muscle dataset (20 slices) in each of the programmes shown in seconds. It was performed on Toshiba laptop with Intel® Core i7‐5500U CPU, 2.40 GHz, 16 GB RAM and 64 bit

		Fiji	Amira	MIB
Foetal	Nucleus	6.29	3.21	2.12
	mitochondria	9.14	9.22	26.44
Adult	Nucleus	10.52	5.35	3.07
	mitochondria	37.14	4.28	62.39

### Segmentation of cellular structures

**Figure 3:**
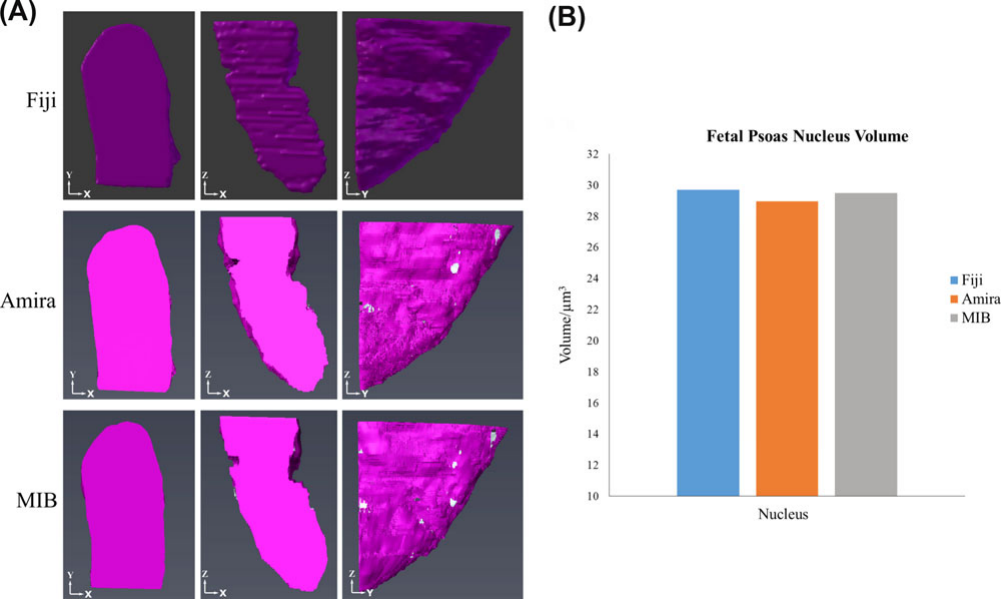
Examples of digital reconstruction of nuclear volume. (A) Reconstructions of the nucleus from foetal psoas at different orientations from the segmentations performed in Fiji, Amira and MIB. The reconstructions of the nuclei show no differences between the different programmes. (B) Volume measurements of the nucleus from each of the programmes, which again are similar between each of the programmes used to reconstruct the nucleus.

**Figure 4:**
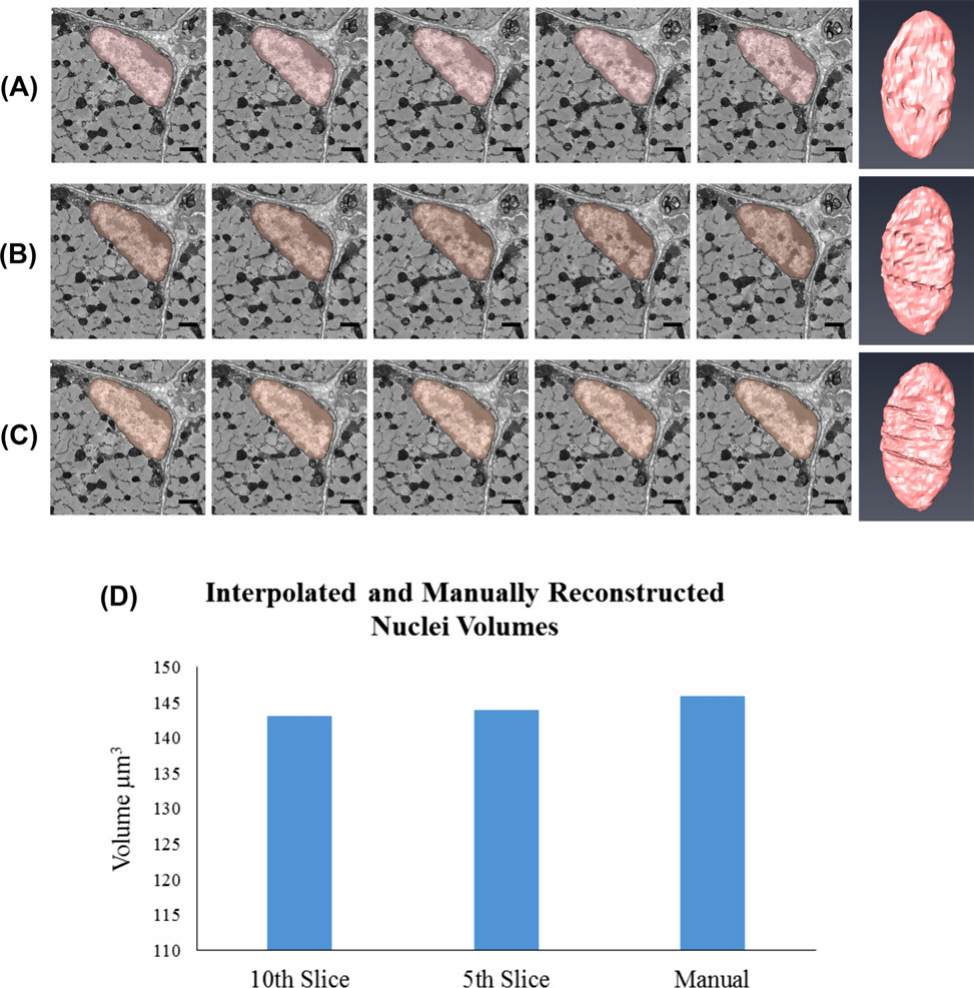
Analysis of accuracy of interpolation method for segmentation. (A), (B) and (C) each show five images with the nucleus segmented and the final reconstruction of the nucleus, from late foetal psoas all performed in MIB. (A) Shows the nucleus segmented using interpolation when every 10th slice has been manually segmented, (B) from every fifth slice and (C) is a nucleus which has been segmented manual. In the images, there appear to be small differences between the segmentations, either the selection has not reached the boundary or goes over it. In the reconstructions, the nuclear folds are not as detailed in (A) and (B) when compared to the manual reconstruction in (C). (D) Volumes from each of the segmentations. The images are cropped from a total image size of 3000 × 3000 pixels taken at 2k× magnification and 13 nm resolution. All scale bars are 1 μm.

The nucleus of the cell was segmented first. This was done manually in Fiji and with the use of interpolation in Amira and MIB. Visually, the 3D models of the nuclei are similar and the volumes are also similar between each of the programmes (Fig. 3). Although using interpolation speeds up the segmentation process, errors did occur on the slices that were interpolated and correcting these added to the time taken to segment. Further analysis was performed to test the accuracy of the interpolation in MIB, comparing the uncorrected interpolated nucleus to a manually segmented one (Fig. 4). There was a differing level of detail between each of the models. Specifically, the folds of the nuclear membrane were not as detailed in the interpolated models as in the manually segmented one. However, the volumes of the nuclei from the quantification analysis were similar, with only 1.98% difference between the corrected and the every 10th slice (1.37% difference between the corrected and the every 5th slice). Therefore, for bulk quantification analysis, corrections may not be required. If, however, finer details are required for qualitative analysis, the interpolation errors will need to be corrected.

**Figure 5:**
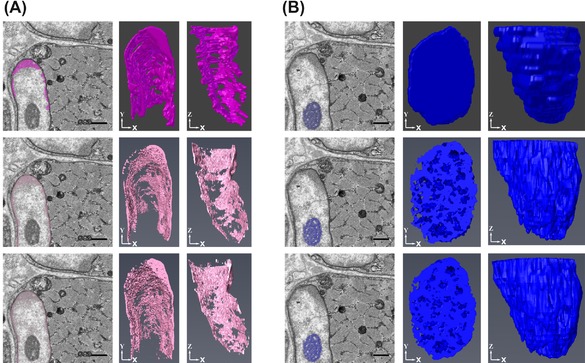
Examples of reconstruction of chromatin and nucleoli. (A) Three single snapshots from the data series with the chromatin segmented and the reconstructions at different orientations performed in each of the image programmes, Fiji, Amira and MIB. (B) Three single snapshots from the data series with the nucleolus segmented and reconstructions of the nucleolus from the three programmes. The nucleoli reconstructed in Amira and MIB show more detail of the ‘web‐like’ appearance of the nucleolus. All scale bars are 1 μm.

After the nucleus, the darker and dense chromatin within the nucleus was segmented. The segmentation and the final reconstruction can be seen in Figure 5(A). Thresholding was used in MIB and Amira to select the darker pixels that correspond to the chromatin. During the thresholding of the chromatin in both Amira and MIB, there were some errors in the selection. In this case, the thresholding was not restricted to a specific structure, and the nuclear boundary was also selected. In order for the thresholding procedure to be effective, there also needs to be sufficient contrast within the images. Manual alterations can be made after thresholding to ensure that the correct selection is made although one has to be careful not to introduce user bias in object selection.

The next feature to be segmented was the nucleolus. The segmentation had to be performed in this order due to the nature of the segmentation in MIB and Amira. As mentioned earlier, in these two programmes, the pixels can only be assigned to a single structure. After the initial segmentation of the nucleus, all pixels were assigned to it. Then, when the chromatin is segmented, the thresholded pixels were reassigned from the nucleus to the chromatin. However, when the chromatin was thresholded, the nucleolus was also selected, as the pixels are of a similar contrast. By segmenting the nucleolus after the chromatin, the pixels were reassigned to the nucleolus. This has to be kept in mind whenever segmenting a larger structure (nucleus) and the inner detail of it (chromatin and nucleoli) when using programmes such as Amira and MIB.

The segmented image and reconstruction of a nucleolus is shown in Figure 5(B). As seen in the raw images, the nucleoli appeared in a range of contrasts, with light and dark pixels. When thresholded, only the darker pixels were selected, which caused gaps in the model, whereas when it was manually segmented, the entire nucleolus area was segmented. This is one example of when thresholding can be used to show intricate internal structures of an object.

**Figure 6:**
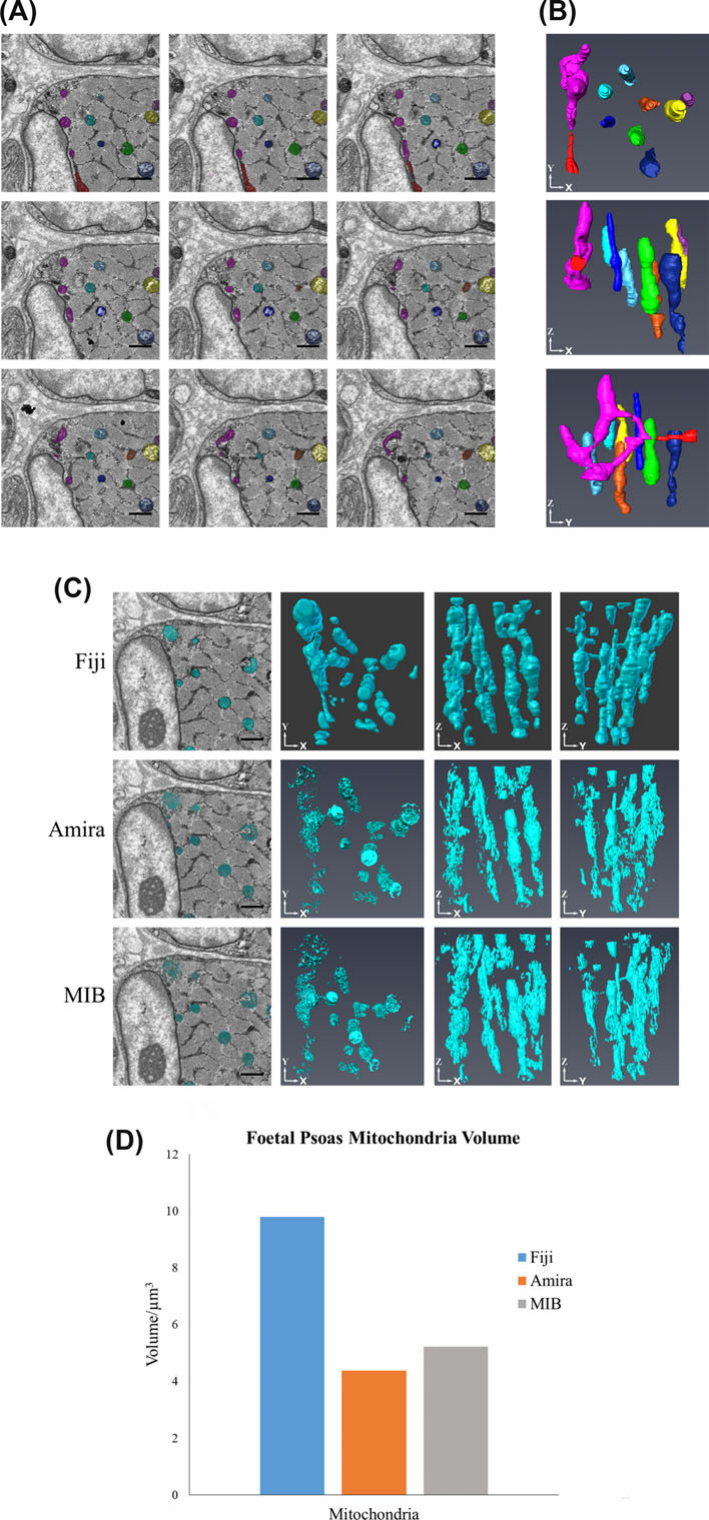
Examples showing the complex morphology of mitochondria. (A) Nine consecutive images from the late foetal psoas (viewed from left to right). The mitochondria have been segmented individually in different colours, in MIB, and their corresponding 3D reconstructions, from Amira, can be seen in (B). Showing how mitochondria change over each 70 nm slice, requiring observation from the user to ensure that the correct structure is selected. All scale bars are 1 μm. (C) Three single snapshots from the data series with the mitochondria segmented and the reconstructions of the mitochondria at different orientations. The mitochondria, reconstructed in Amira and MIB, appear broken due to the selection method used. (D) Volume measurements of the mitochondria, which show that there is a large difference between the Fiji segmentation and the Amira and MIB segmentations, due to the broken appearance seen in the reconstructions. The images were taken at 12k× magnification, 7 nm resolution and an image size of 1024 × 1024 pixels All scale bars are 1 μm.

Once the nuclei and associated structures were completed, the mitochondria were segmented. The decision was made not to interpolate the mitochondria due to their small size and complex nature, as highlighted in Figures 6(A) and (B). Instead, the mitochondria were thresholded in Amira and MIB. On comparing, the three models there were differences (Fig. 6C). Mitochondria consist of a range of contrasts and in the raw images, the detail of the cristae (appearing as dark inner membranes) can be seen. When thresholded, only the darker pixels, the outer membrane and cristae, were selected, which gave the thresholded models a broken appearance. However, the location and arrangement of the mitochondria could still be discerned and with further user input (e.g. using the fill feature), the appearance was improved. However, this is further evidence that the thresholding tool is case‐dependent and often cannot be relied upon on its own but should be used in conjunction with other segmentation tools.

**Figure 7:**
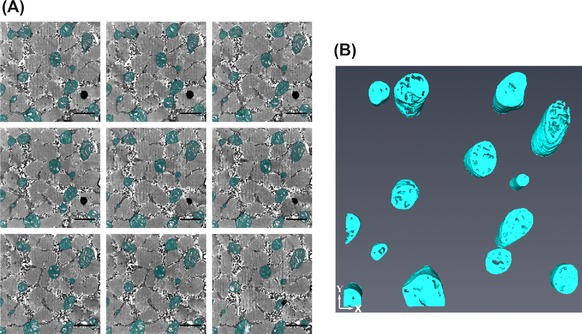
Digital reconstruction of mitochondria from a dataset with thinner sectioning and higher magnification. (A) Nine consecutive images (viewed from left to right) from a larger dataset from the foetal psoas muscle. The images were taken at high magnification (18k×), high resolution (5 nm) and the block was sliced at 40 nm section thickness. (B) The subsequent reconstructions from a portion of the total stack to highlight the reconstruction of the cristae of the mitochondria using the thresholding tool in MIB and Amira to reconstruct the mitochondria. Scale bar is 1 μm.

A second dataset was taken at a higher resolution, higher magnification and thinner section thickness, specifically to attempt to reconstruct the mitochondria with greater detail. Figure 7 presents the results from the reconstruction of these datasets, showing that it was possible for the cristae of the mitochondria to be reconstructed in detail. However, importantly, quantitative analysis of the thresholded mitochondria resulted in a smaller volume than the manually segmented mitochondria, due to only the darker pixels being segmented. Therefore, if quantification of mitochondrial volume is required, a manual method will have to be used to ensure that correct measurements are made.

**Figure 8:**
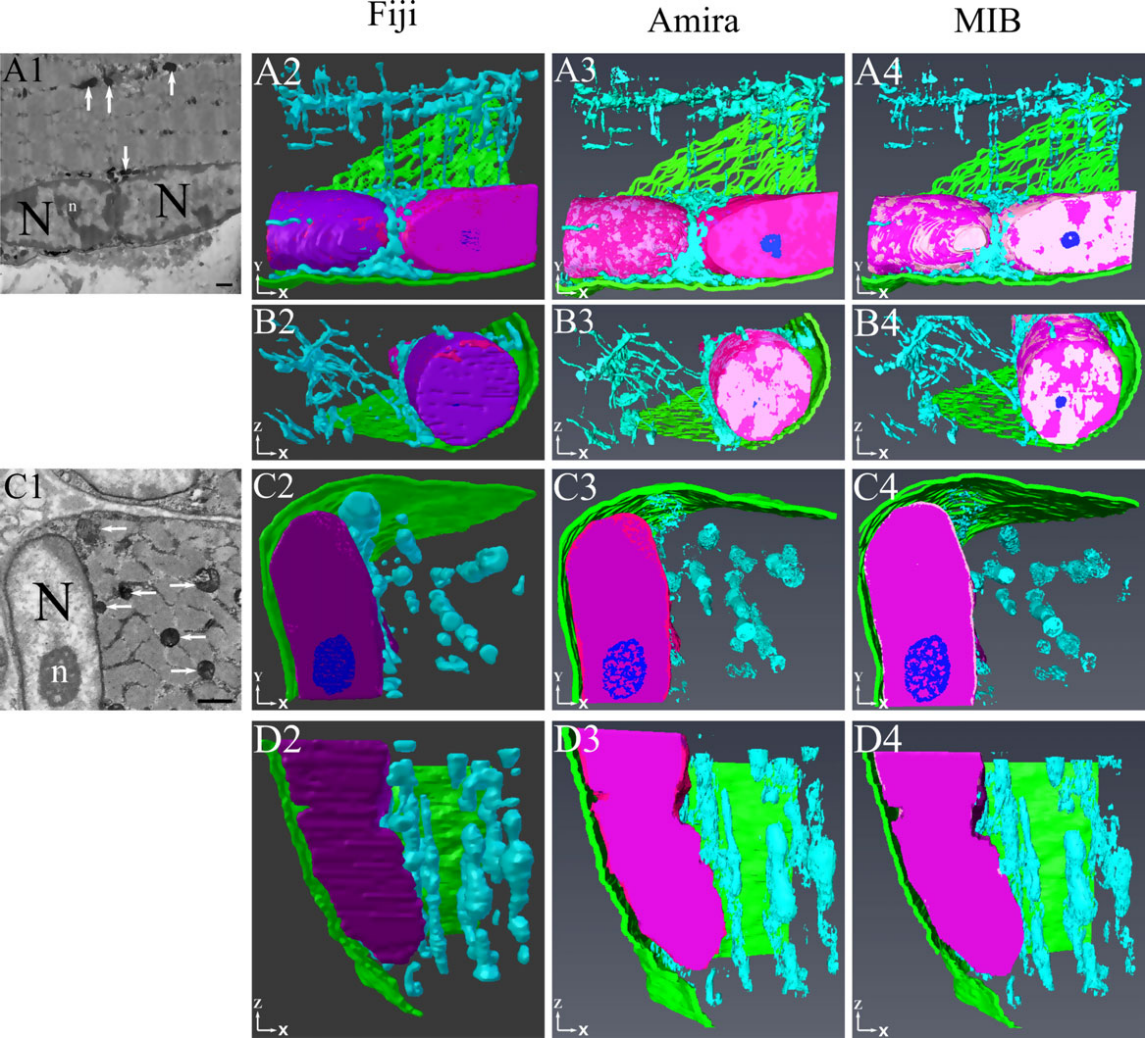
Examples of 3D reconstructions of segmented structures. This diagram depicts examples of the assembled reconstructions of all segmented features from two separate skeletal muscle SBF‐SEM datasets. Rows (A) and (B) show results from adult soleus muscle (from X serial sections; panel A1 indicates a snapshot SBF‐SEM image). Rows C and D show results from foetal psoas muscle (from X serial sections; panel B1 indicates a snapshot SBF‐SEM image). (C) and (D) The following features are colour‐coded in the reconstructions: mitochondria, light blue; nuclei, dark pink/purple; chromatin, light pink; nucleoli, dark blue; plasmalemma, green. All scale bars are 1 μm.

**Figure 9:**
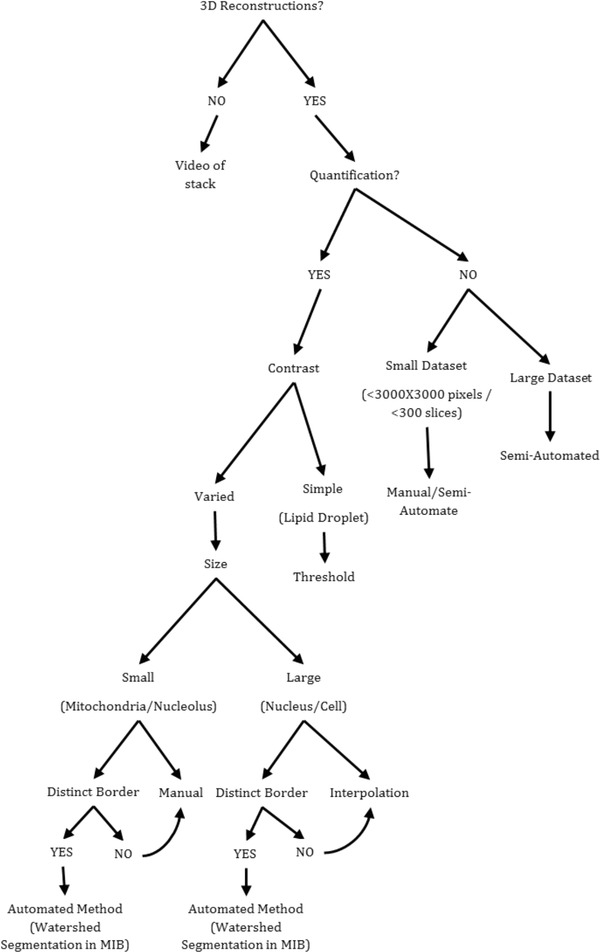
Proposed workflow to aid in decision making when choosing appropriate segmentation methods for analysis of SBF‐SEM data. The majority of these segmentation methods can be used in MIB and Amira, with some exceptions, the watershed segmentation which is not shown in this paper. The decision to use either MIB or Amira to perform the segmentations will depend on user preference and access to the software, as previously shown, the two programmes yield similar results.

The final reconstructions incorporating all segmented features of the skeletal muscle are shown in Figure 8. Although the general appearances of the models were similar in each of the programmes, there were some differences caused by the methods used to segment the structures of interest. For example, the mitochondria were more fragmented in the MIB and Amira models as they had been thresholded compared to the more dense structures seen with Fiji's manual segmentation. The devised workflow from these analyses is shown in Figure 9.

### Validation

The workflow was then implemented onto two other tissue types: guinea pig cardiac muscle and a region of the optic lobe from locust brain. This was done to test the workflow and validate that it was applicable to other tissues and structures.

**Figure 10:**
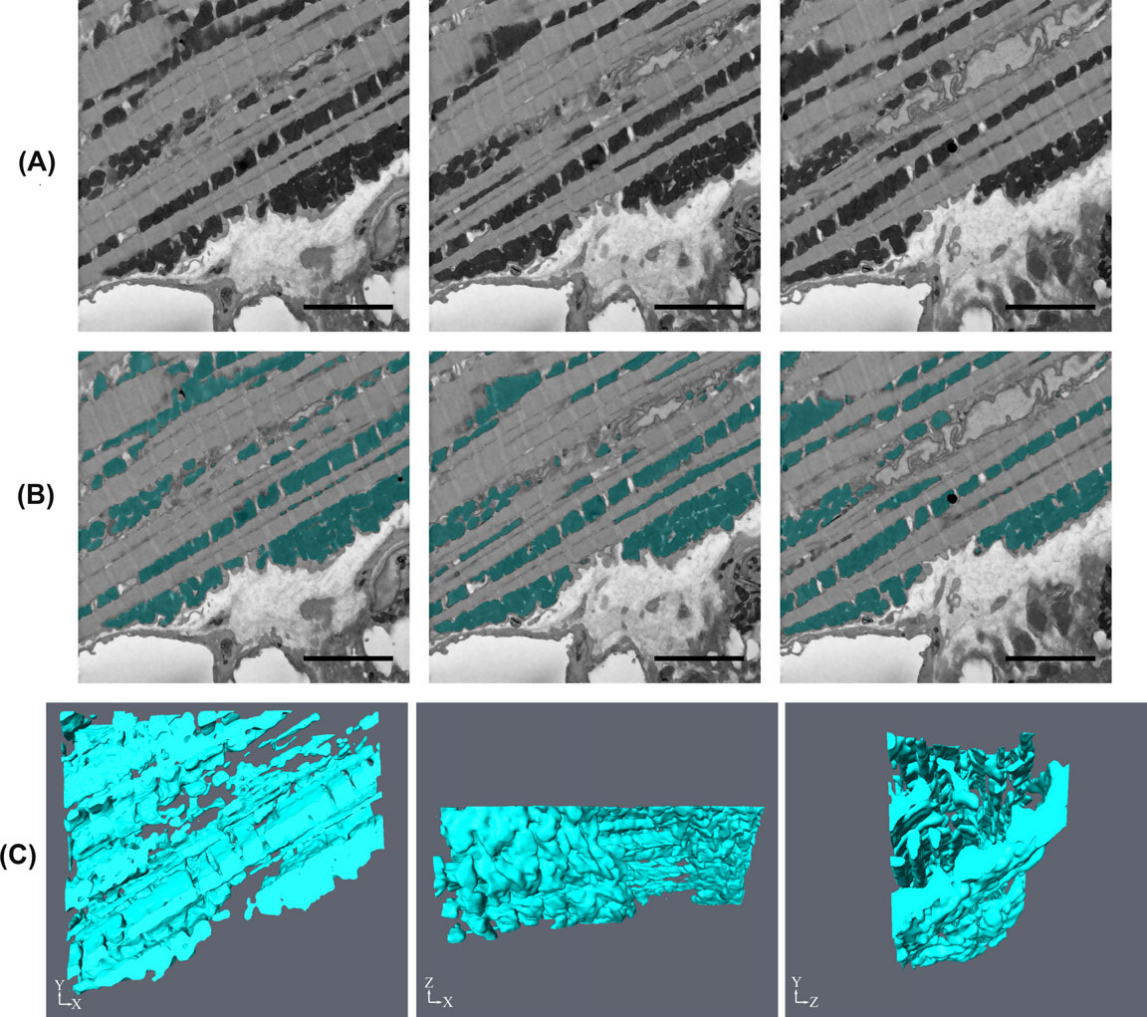
Segmentation and reconstruction of mitochondria from cardiac muscle. (A) Shows three raw images that are five slices apart from each other, (B) is the same raw images with the segmentation of the mitochondria shown and (C) is the subsequent reconstructions at different orientations. The images were taken at 5k× magnification, 18 nm resolution and an image size of 1024 × 1024 pixels. Scale bars are 1 μm.

The mitochondria in the cardiac muscle were dark and dense, with high contrast to surrounding features (Fig. 10A). It had a different appearance when compared to the mitochondria in the skeletal muscle, which, as shown already, exhibited varied contrasts. By following the workflow (3D Reconstruction>Yes>Quantification>Yes>Contrast>Simple>Threshold), it was determined that a form of thresholding, either b/w or the magic wand in MIB or Amira, could be used to segment the cardiac mitochondria. This was done and the segmentation and subsequent reconstructions are shown in Figures 10(B) and (C).

**Figure 11:**
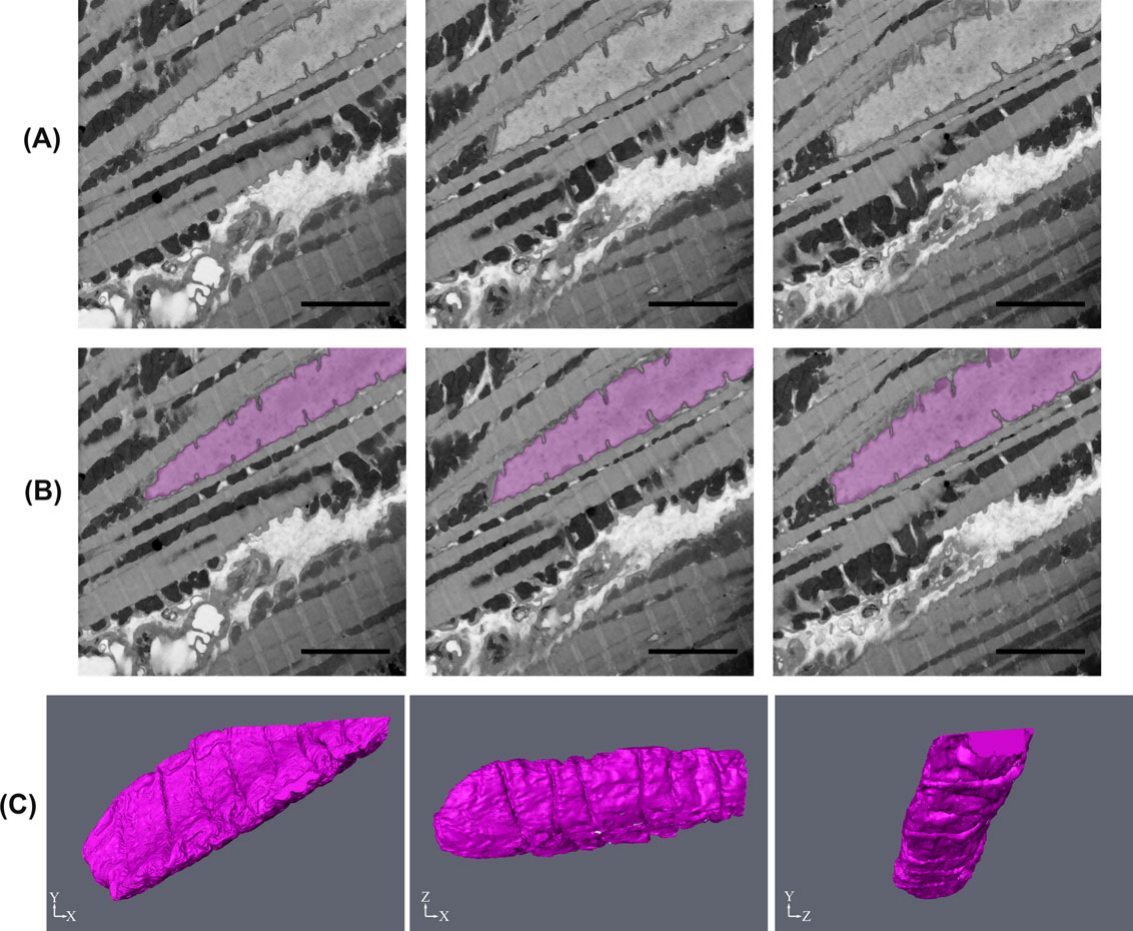
Segmentation and reconstruction of nucleus from cardiac muscle. (A) Shows three raw images that are five slices apart from each other, (B) is the same raw images with the segmentation of the nucleus shown and (C) is the subsequent reconstructions at different orientations. The images were taken at 5k× magnification, 18 nm resolution and an image size of 1024 × 1024 pixels. Scale bars are 1 μm.

For the cardiac muscle nucleus, the same segmentation procedure was used as for the skeletal muscle. The workflow was (3D Reconstruction>Yes>Quantification>Yes>Contrast>Varied>Size> Large>Distinct Border>Yes>Automated Method (Watershed Segmentation in MIB)). The dataset was composed of only 100 slices, image size 1024 × 1024 pixels, and the nucleus was only present for a portion of the stack. As automated segmentation can be a time‐consuming method due to computational demands, it is not efficient for small objects or datasets. Thus, the decision was made to use manual segmentation combined with interpolation. So, the nucleus was manually segmented over every fifth slice, interpolated in between and any errors manually corrected (Fig. 11).

**Figure 12:**
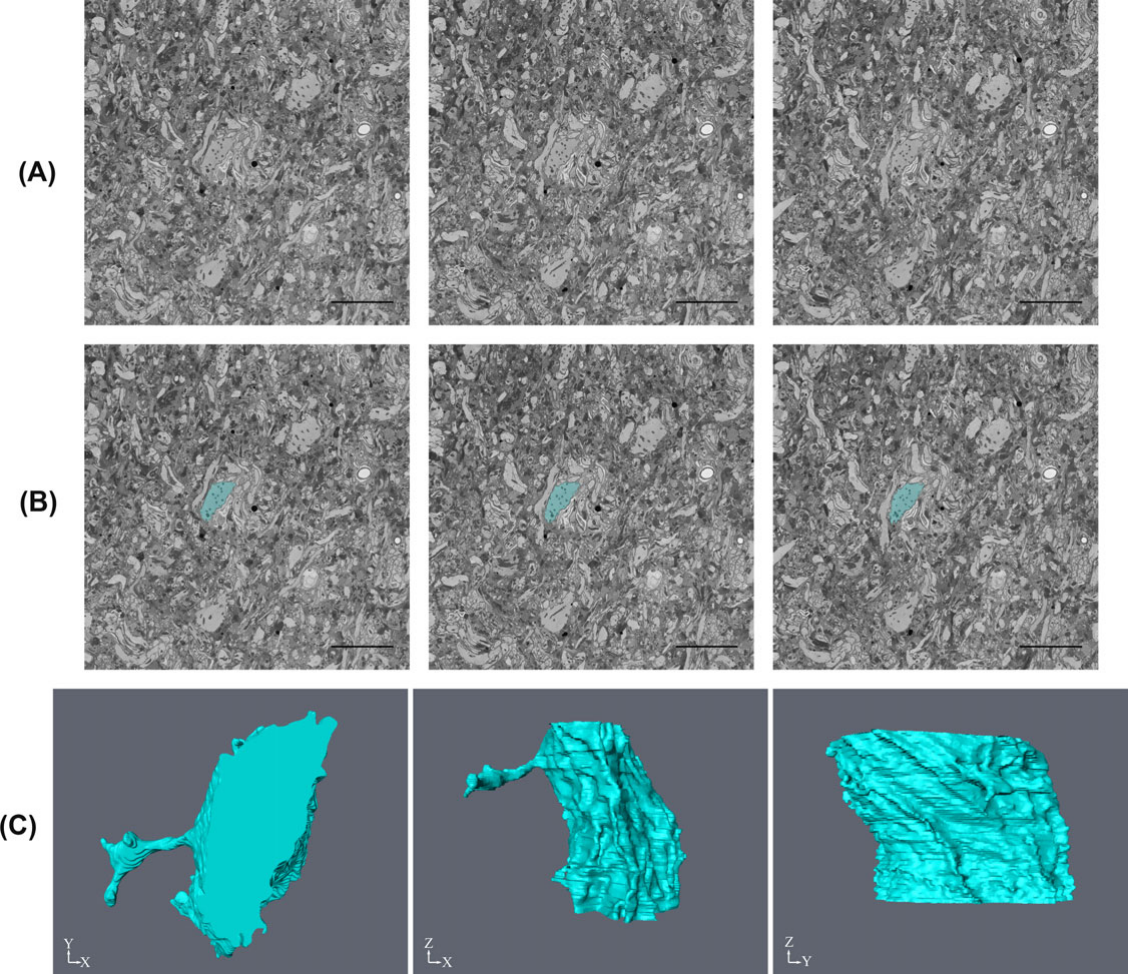
Segmentation and reconstruction of the LGMD2 neuron from the optic lobe of the locust. (A) Shows three raw images that are five slices apart from each other, (B) is the same raw images with the segmentation of the LGMD2 shown and (C) is the subsequent reconstructions at different orientations. The images were taken at 1.5k× magnification, 9 nm resolution, section thickness of 60 nm and an image size of 6000 × 6000 pixels. Scale bars are 5 μm.

A similar dilemma was found in the dataset from the locust optic lobe of the Lobula Giant Movement Detector 2 (LGMD2) neuron. This tissue was vastly different from the skeletal and cardiac muscle already shown, as it was densely packed with a variety of cells. The dataset analysed (100 slices at 6000 × 6000 pixels) was also much larger than the muscle data. The aim, on this occasion, was rather different: to segment out an entire dendrite and the following workflow was followed 3D Reconstruction>Yes>Quantification>Yes>Contrast>Varied>Size> Large>Distinct Border>Yes>Automated Method (Watershed Segmentation in MIB). As mentioned earlier, this method takes time but there is a semiautomated version of the watershed segmentation combined with the brush tool in MIB, as described in the methods. Using this method, the dendrite was selected and selection was repeated every fifth slice and interpolation was used (Fig. 12). This tool could be used when analysing nuclei, however, it is best used on larger and more complex structures that would take time to manually segment, such as large dendrites like the LGMD2.

## Discussion

Serial block face SEM is a powerful tool for cellular examination and, when combined with image analysis software, can provide detailed qualitative and quantitative data. Since its release in 2004, the use SBF‐SEM has steadily grown. In 2016, there were 39 articles published and so far in 2017, there have been 24 publications using this technique. The articles were found by searching ‘serial block face scanning EM’ in PubMed (https://www.ncbi.nlm.nih.gov/pubmed/) and only selecting those that use the technique for their research, not a review of the technique. With this burgeoning interest, there has been an increased demand for training in the analysis of data. This has drawn attention to the need for clarity and consistency in the reporting of methods of data analysis and interpretation, both qualitative and quantitative. However, getting started with the software is not easy for new researchers and there is a risk they will underutilize their data. Here, we have explained the terminology of many analytical features found in the programmes and provided step‐by‐step protocols to instruct users.

We have compared manual and semiautomated segmentation methods in order to help researchers chose the best options for their analysis. The results show that the semiautomated methods are less time‐consuming but are not always accurate, as shown by the quantification results of the mitochondria segmentations. However, this segmentation was performed with the thresholding tools, which work best on structures of a single contrast that are distinct from their surroundings. Thresholding can be a useful way to highlight the finer details of structures, such as the cristae of the mitochondria, the web‐like appearance of the nucleolus and the chromatin within the nucleus. In all cases, these semiautomated tools require some form of manual input and manual correction. A prime example of this is when using interpolation, which is best suited to larger structures that do not change much over each slice, like the nucleus of a cell, a whole cell or a large portion of the tissue. To optimize results, each object of interest should be assessed individually in terms of the segmentation method. The tools mentioned in this paper, whilst commonly used, are just some that are available. There are many other possibilities some of which have been briefly mentioned in this paper (e.g. creation of masks, smoothening, smart watershed and graph cut segmentation) for users more familiar with the programmes. Using a range of tools to efficiently and accurately segment, multiple structures in a sample will give better results than trying to use a ‘one‐method‐fits‐all’ approach.

Although the results shown here are derived mostly from guinea pig skeletal muscle, the described approaches have been repeated on other types of tissue (guinea pig cardiac muscle and locust optic lobe), which validate the findings shown. From the results, a workflow was devised, Figure 9, to aid researchers new to image analysis. It provides recommendations for segmentation tools based on a variety of factors, such as the contrast and size of the structure, presence of any membrane boundaries, the size of the dataset and most importantly the objective of the analysis. The result can be qualitative, quantitative or both and knowing which is required prior to analysis is important. Knowing the objective is not only important for the image analysis but also prior to that, when the datasets are collected. For example, if low‐resolution images are collected to reconstruct a whole cell, it is no good deciding after collection that the organelles should also be reconstructed, as they may have insufficient clarity. The resolution of the image collection should be determined by the smallest structure likely to be of interest. However, although decisions on how to collect data in pursuit of biological questions are made at the time of SBF‐SEM scanning, the outcomes are only revealed upon viewing the serially collected digital images. Indeed, this is one of the key benefits of SBF‐SEM. Therefore, if tissue is plentiful, an iterative approach to SBF‐SEM data collection and analysis can be beneficial in revealing much new biological information from complex cell/tissue structures.

Before the data collection starts, the settings of the SBF‐SEM have to be adjusted to the needs of the researcher, for example, the accelerating voltage and pressure. The voltage and pressure are closely linked, if one is dropped, the other has to be dropped; otherwise, there is a loss of contrast to the image. We have found that at a lower kilovolts and lower pascals, the amount of charging by the electron beam is reduced and imaging at settings as low as 2.5 kV and a pressure between 20 and 25 pa can yield excellent results. High vacuum can also be used to give high‐resolution images; however, it is limited to dense tissues, as the electron beam affects the resin, the image ‘jumps’ and the datasets have to be aligned. However, these parameters can be tissue‐ and machine‐dependent. It is likely that different versions of SBF‐SEM systems can operate at high vacuum better than others and we recommend researchers test a combination of kilovolts and pascals to find one that suits their tissue and their objective.

By adjusting the data collection parameters – section thickness, magnification and number of pixels – the resulting images will have different resolution, thus altering the appearance of the final reconstructions. A thinner section thickness will result in a more detailed reconstruction, as smaller changes in structures will be imaged. A higher resolution results in a clearer image, so a better distinction between cellular structures and higher detail of the structures is achieved. The resolution, magnification and number of pixels are all linked. A higher magnification results in a higher resolution image but this decreases the field of view. To maintain the same field of view but still increase resolution, the number of pixels can be increased. However an increase in pixel number also increases the resulting image file size, which can be difficult to handle without a high‐powered computer. For the majority of this analysis the datasets were <3000 × 3000 pixels and <300 slices, so a laptop with 16 GB RAM and 2.4 GHz processing speed could cope. However for larger datasets (e.g. >3000 × 3000 pixels and >400 slices) a more high powered computer, such as with 64 GB RAM and 3.2 GHz processing speed may be needed. In addition, if the time taken to collect an image is very high, the electron dose can have a detrimental effect on the resin and cause subsequent sectioning artefact. An alternative would be multiple regions of interest (ROIs) at a higher magnification but lower pixel number. However, ROIs that overlap might be affected by the increased exposure to the electron beam, as the areas are scanned repeatedly. Thus, some compromise may be required to balance the need for high‐resolution images but also images free from sectioning and imaging artefacts.

We have provided a workflow to recommend segmentation tools and also a detailed step‐by‐step protocol for four programmes, one of which is recently developed (MIB). The aim is to provide a resource for researchers to refer to when starting their analysis. Although there have been several publications using SBF‐SEM, the detail about the segmentation methods is limited. We carried out a survey of the recent literature (Supplementary file S6) where SBF‐SEM has been utilized and found that although all papers stated which programme they had used, only around half stated the specific tools utilized. Of these, the majority simply stated the type of segmentation and only a small number of the articles described the segmentation process in detail (Supplementary file S6). From the literature survey, it was also apparent that different terms were used to describe the segmentation tools, for example, manual segmentation was sometimes referred to ‘by hand’ and thresholding as ‘intensity‐based’ (Meyer *et al*., 2017) or ‘contrast‐based selection’ (Pinali & Kitmitto, 2014). This could lead to confusion for those new to this type of analysis. An article by Borrett and Hughes (2016) reviewed earlier publications and they also noted that there was often a lack of information given in articles on the methods used to analyse data from SBF‐SEM. From the analysis of the literature, manual segmentation appears to be the preferred method. However, this could be from a lack of knowledge or confidence rather than it being the most suitable method of segmentation. For a detailed analysis of the different software programmes and their functionalities, the reader is directed to Kittelmann *et al*. (2016).

In addition, we recognize that this is a fluid research environment where advances in computational analysis tools are rapid. Therefore, our provision of openly accessible raw datasets enables researchers who are developing novel/improved analysis approaches to compare the functionality of new tools with those used here. All researchers should be encouraged to deposit future SBF‐SEM datasets at https://www.ebi.ac.uk/pdbe/emdb/empiar/, or similar open‐access repositories. We have not only provided detailed step‐by‐step protocols but also videos to run alongside these protocols. We have found that when teaching others to use new programmes, it is much easier for them to learn with textual and visual explanations available in tandem.

In conclusion, SBF‐SEM is a powerful tool for analysing cellular structures with high resolution in *x*–*y*–*z* planes. However, current publications do not always give enough information on the data analyses involved and with the myriad of programmes and tools available, it can be a daunting task for new researchers to train themselves. By following a logical workflow such as that provided here, it is possible to obtain qualitative and quantitative data on multiple structures from a single dataset, maximizing output from valuable tissue.

## Supporting information


**S1. Image Processing Fiji**. Converting from DM3 to Tiff.

**S2. Fiji/TrakEM2**. Image analysis in Fiji and TrakEM2.

**S3. Blender**. How to use Blender to show reconstructions.

S4. Microscopy Image Browser (MIB). Image analysis in MIB.

**S5. Amira**. Image analysis in Amira.

**S6. Analysis Tools for SBG‐SEM Datasets in Recent Literature**. Articles published between 2015 and 2017 including SBF‐SEM experimentation were assessed for the detail they provided on the segmentation process used, including any software programmes and specific tools.
