# Supplementary material for: A guide to analysis and reconstruction of serial block face scanning electron microscopy data
Source: J Microsc. 2018 Jan 15;270(2):217–34. doi: 10.1111/jmi.12676 (PMC5947172; doi:10.1111/jmi.12676)
Supplement: Supplementary file 6 — S6. Analysis Tools for SBG‐SEM Datasets in Recent Literature. Articles published between 2015 and 2017 including SBF‐SEM experimentation were assessed for the detail they provided on the segmentation process used, including any software programmes and specific tools. [file JMI-270-217-s006.docx]

| **Paper** | **Software** | **Segmentation Tool** |
| --- | --- | --- |
| *Bellesi et al, 2015* | Fiji (TrakEm2) | Manual |
| *Biazik et al, 2015a* | IMOD | Manual |
| *Biazik et al, 2015b* | IMOD | Manual |
| *Bohórquez et al, 2015* | Imaris | Manual |
| *Breidenbach et al, 2015* | IMOD | Interpolation and Manual |
| *Chavan et al, 2015* | Fiji(TrakEM2) | Not Stated |
| *Gluenz et al, 2015* | IMOD and Amira | Manual and Thresholding |
| *Hammer et al, 2015* | Fiji(TrakEM2) | Manual |
| *Hanlon et al, 2015* | Amira | Not Stated |
| *Hondow et al, 2015* | Fiji and Imaris | Not Stated |
| *Ichimura et al, 2015* | Amira | Not Stated |
| *Jungreuthmayer et al, 2015* | ImageJ (BoneJ) | Thresholding |
| *Kleesattel, Crish and Inman, 2015* | Reconstruct | Not Stated |
| *Mourik et al, 2015* | Amira | Manual and Thresholding |
| *Neves and Reichert, 2015* | Fiji(TrakEM2) | Manual |
| *Ou et al, 2015* | IMOD | Manual and Interpolation |
| *Pfeifer et al, 2015* | Amira | Manual |
| *Pinali et al, 2015* | IMOD and Fiji | Not Stated |
| *Shomorony et al, 2015* | Amira | Not Stated |
| *Wernitznig et al, 2015* | Amira | Manual |
| *Yamada et al, 2015* | ImageJ and Amira | Manual and Automated |
| *Booth et al, 2016* | Amira | Masking, Thresholding, Magic Wand and Blow Tools |
| *Büsse et al, 2016* | Amira | Not Stated |
| *Feng et al, 2016* | Fiji and IMOD | Manual and Interpolation |
| *Han et al, 2016* | IMOD | Manual |
| *Jakob et al, 2016* | IMOD | Not Stated |
| *Kaiser et al, 2016* | Fiji | Not Stated |
| *Kaji et , 2016* | Imaris | Not Stated |
| *Lewis et al, 2016* | Amira | Not Stated |
| *Mukherjee et al, 2016* | Fiji (TrakEM2) | Manual |
| *Nguyen et al, 2016* | Fiji(TrakEM2) and Amira | Not Stated |
| *Nkwe et al, 2016* | Amira and Fiji (TrakEM2) | Manual |
| *Ochs et al, 2016* | Not Said | Not Stated |
| *Pipkin et al, 2016* | IMOD and Fiji | Manual |
| *Randles et al, 2016* | IMOD | Manual |
| *Rind et al, 2016* | Amira | Not Stated |
| *Sakaguchi et al, 2016* | Amira | Manual and Automated |
| *Shami et al, 2016* | ImageJ and IMOD | Manual |
| *Smith et al, 2016* | Synapse Web Reconstruct | Not Stated |
| *Stucki et al, 2016* | UCSF Chimera and IMOD | Not Stated |
| *Thai et al, 2016* | Fiji (TrakEM2) and Amira | Not Stated |
| *Vincent et al, 2016* | IMOD | Manual |
| *Wanner et al, 2016* | PyKnossos | Manual |
| *Wernitznig et al, 2016* | Amira and own tool | Semi-Automated and Manual |
| *Agrawal et al, 2017* | Amira | Not Stated |
| *Bellesi et al, 2017* | Fiji (TrakEM2) | Manual |
| *Cabezón et al, 2017* | Imaris | Not Stated |
| *Chai et al, 2017* | Reconstruct | Not Stated |
| *Chen et al, 2017* | Avizo | Not Stated |
| *Chung et al, 2017* | Avizo | Intensity-based Thresholding |
| *Colman et al, 2017* | IMOD and Fiji | Manual and Thresholding |
| *Giarmarco et al, 2017* | Fiji (TrakEM2) | Manual |
| *Gillies et al, 2017* | IMOD | Manual |
| *Hoover et al, 2017* | Imaris | Manual |
| *Hughes et al, 2017* | Amira | Manual and Thresholding |
| *Ichimura et al, 2017* | Amira | Not Stated |
| *Martin et al, 2017* | Fiji (TrakEM2) | Manual |
| *Meyer et al, 2017* | Imaris | Intensity Thresholding and Size Filtering |
| *Nixon et al, 2017* | Amira | Manual |
| *Palaiologou et al, 2017* | Amira | Thresholding |
| *Pinali et al, 2017* | IMOD | Not Stated |
| *Plachno et al, 2017* | Imaris | Manual |
| *Sai et al, 2017* | Amira | Manual |
| *Svensson et al, 2017* | Amira | Manual |
| *Szczesny et al, 2017* | Amira | Region Growing Algorithm |
| *Walker et al, 2017* | Fiji (TrakEM2) | Manual |
| *White et al, 2017a* | Amira | Not Stated |
| *White at al, 2017b* | Amira | Not Stated |

**References**

Agrawal, S. A., Burgoyne, T., Eblimit, A., Bellingham, J., Parfitt, D. A., Lane, A., Nichols, R., Asomugha, C., Hayes, M. J., Munro, P. M., Xu, M., Wang, K., Futter, C. E., Li, Y., Chen, R. & Cheetham, M. E. (2017) REEP6 deficiency leads to retinal degeneration through disruption of ER homeostasis and protein trafficking. *Hum Mol Genet,* **26,** 2667-2677.

Bellesi, M., de Vivo, L., Chini, M., Gilli, F., Tononi, G. & Cirelli, C. (2017) Sleep Loss Promotes Astrocytic Phagocytosis and Microglial Activation in Mouse Cerebral Cortex. *J Neurosci,* **37,** 5263-5273.

Bellesi, M., de Vivo, L., Tononi, G. & Cirelli, C. (2015) Effects of sleep and wake on astrocytes: clues from molecular and ultrastructural studies. *BMC Biol,* **13,** 66.

Biazik, J., Vihinen, H., Anwar, T., Jokitalo, E. & Eskelinen, E. L. (2015a) The versatile electron microscope: an ultrastructural overview of autophagy. *Methods,* **75,** 44-53.

Biazik, J., Yla-Anttila, P., Vihinen, H., Jokitalo, E. & Eskelinen, E. L. (2015b) Ultrastructural relationship of the phagophore with surrounding organelles. *Autophagy,* **11,** 439-451.

Bohorquez, D., Haque, F., Medicetty, S. & Liddle, R. A. (2015) Correlative Confocal and 3D Electron Microscopy of a Specific Sensory Cell. *J Vis Exp***,** e52918.

Booth, D. G., Beckett, A. J., Molina, O., Samejima, I., Masumoto, H., Kouprina, N., Larionov, V., Prior, I. A. & Earnshaw, W. C. (2016) 3D-CLEM Reveals that a Major Portion of Mitotic Chromosomes Is Not Chromatin. *Mol Cell,* **64,** 790-802.

Breidenbach, A. P., Aschbacher-Smith, L., Lu, Y., Dyment, N. A., Liu, C. F., Liu, H., Wylie, C., Rao, M., Shearn, J. T., Rowe, D. W., Kadler, K. E., Jiang, R. & Butler, D. L. (2015) Ablating hedgehog signaling in tenocytes during development impairs biomechanics and matrix organization of the adult murine patellar tendon enthesis. *J Orthop Res,* **33,** 1142-1151.

Busse, S., Hornschemeyer, T. & Fischer, C. (2016) Three-dimensional reconstruction on cell level: case study elucidates the ultrastructure of the spinning apparatus of Embia sp. (Insecta: Embioptera). *R Soc Open Sci,* **3,** 160563.

Cabezon, I., Auge, E., Bosch, M., Beckett, A. J., Prior, I. A., Pelegri, C. & Vilaplana, J. (2017) Serial block-face scanning electron microscopy applied to study the trafficking of 8D3-coated gold nanoparticles at the blood-brain barrier. *Histochem Cell Biol,* **148,** 3-12.

Chai, H., Diaz-Castro, B., Shigetomi, E., Monte, E., Octeau, J. C., Yu, X., Cohn, W., Rajendran, P. S., Vondriska, T. M., Whitelegge, J. P., Coppola, G. & Khakh, B. S. (2017) Neural Circuit-Specialized Astrocytes: Transcriptomic, Proteomic, Morphological, and Functional Evidence. *Neuron,* **95,** 531-549 e539.

Chavan, V., Willis, J., Walker, S. K., Clark, H. R., Liu, X., Fox, M. A., Srivastava, S. & Mukherjee, K. (2015) Central presynaptic terminals are enriched in ATP but the majority lack mitochondria. *PLoS One,* **10,** e0125185.

Chen, B., Yusuf, M., Hashimoto, T., Estandarte, A. K., Thompson, G. & Robinson, I. (2017) Three-dimensional positioning and structure of chromosomes in a human prophase nucleus. *Sci Adv,* **3,** e1602231.

Chung, K. F., Seiffert, J., Chen, S., Theodorou, I. G., Goode, A. E., Leo, B. F., McGilvery, C. M., Hussain, F., Wiegman, C., Rossios, C., Zhu, J., Gong, J., Tariq, F., Yufit, V., Monteith, A. J., Hashimoto, T., Skepper, J. N., Ryan, M. P., Zhang, J., Tetley, T. D. & Porter, A. E. (2017) Inactivation, Clearance, and Functional Effects of Lung-Instilled Short and Long Silver Nanowires in Rats. *ACS Nano,* **11,** 2652-2664.

Colman, M. A., Pinali, C., Trafford, A. W., Zhang, H. & Kitmitto, A. (2017) A computational model of spatio-temporal cardiac intracellular calcium handling with realistic structure and spatial flux distribution from sarcoplasmic reticulum and t-tubule reconstructions. *PLoS Comput Biol,* **13,** e1005714.

Feng, T., Paterson, B. D., Webb, R. & Johnston, S. D. (2016) Three-dimensional reconstruction of black tiger prawn (Penaeus monodon) spermatozoa using serial block-face scanning electron microscopy. *J Morphol,* **277,** 565-574.

Giarmarco, M. M., Cleghorn, W. M., Sloat, S. R., Hurley, J. B. & Brockerhoff, S. E. (2017) Mitochondria Maintain Distinct Ca2+ Pools in Cone Photoreceptors. *J Neurosci,* **37,** 2061-2072.

Gillies, A. R., Chapman, M. A., Bushong, E. A., Deerinck, T. J., Ellisman, M. H. & Lieber, R. L. (2017) High resolution three-dimensional reconstruction of fibrotic skeletal muscle extracellular matrix. *J Physiol,* **595,** 1159-1171.

Gluenz, E., Wheeler, R. J., Hughes, L. & Vaughan, S. (2015) Scanning and three-dimensional electron microscopy methods for the study of Trypanosoma brucei and Leishmania mexicana flagella. *Methods Cell Biol,* **127,** 509-542.

Hammer, S., Monavarfeshani, A., Lemon, T., Su, J. & Fox, M. A. (2015) Multiple Retinal Axons Converge onto Relay Cells in the Adult Mouse Thalamus. *Cell Rep,* **12,** 1575-1583.

Han, B., Newbould, M., Batra, G., Cheesman, E., Craigie, R. J., Mohamed, Z., Rigby, L., Padidela, R., Skae, M., Mironov, A., Starborg, T., Kadler, K. E., Cosgrove, K. E., Banerjee, I. & Dunne, M. J. (2016) Enhanced Islet Cell Nucleomegaly Defines Diffuse Congenital Hyperinsulinism in Infancy but Not Other Forms of the Disease. *Am J Clin Pathol,* **145,** 757-768.

Hanlon, S. D., Behzad, A. R., Sakai, L. Y. & Burns, A. R. (2015) Corneal stroma microfibrils. *Exp Eye Res,* **132,** 198-207.

Hondow, N., Brown, M. R., Starborg, T., Monteith, A. G., Brydson, R., Summers, H. D., Rees, P. & Brown, A. (2016) Quantifying the cellular uptake of semiconductor quantum dot nanoparticles by analytical electron microscopy. *J Microsc,* **261,** 167-176.

Hoover, B., Baena, V., Kaelberer, M. M., Getaneh, F., Chinchilla, S. & Bohorquez, D. V. (2017) The intestinal tuft cell nanostructure in 3D. *Sci Rep,* **7,** 1652.

Hughes, L., Borrett, S., Towers, K., Starborg, T. & Vaughan, S. (2017) Patterns of organelle ontogeny through a cell cycle revealed by whole-cell reconstructions using 3D electron microscopy. *J Cell Sci,* **130,** 637-647.

Ichimura, K., Kakuta, S., Kawasaki, Y., Miyaki, T., Nonami, T., Miyazaki, N., Nakao, T., Enomoto, S., Arai, S., Koike, M., Murata, K. & Sakai, T. (2017) Morphological process of podocyte development revealed by block-face scanning electron microscopy. *J Cell Sci,* **130,** 132-142.

Ichimura, K., Miyazaki, N., Sadayama, S., Murata, K., Koike, M., Nakamura, K., Ohta, K. & Sakai, T. (2015) Three-dimensional architecture of podocytes revealed by block-face scanning electron microscopy. *Sci Rep,* **5,** 8993.

Jakob, M., Hoffmann, A., Amodeo, S., Peitsch, C., Zuber, B. & Ochsenreiter, T. (2016) Mitochondrial growth during the cell cycle of Trypanosoma brucei bloodstream forms. *Sci Rep,* **6,** 36565.

Jungreuthmayer, C., Steppert, P., Sekot, G., Zankel, A., Reingruber, H., Zanghellini, J. & Jungbauer, A. (2015) The 3D pore structure and fluid dynamics simulation of macroporous monoliths: High permeability due to alternating channel width. *J Chromatogr A,* **1425,** 141-149.

Kaiser, G., De Niz, M., Zuber, B., Burda, P. C., Kornmann, B., Heussler, V. T. & Stanway, R. R. (2016) High resolution microscopy reveals an unusual architecture of the Plasmodium berghei endoplasmic reticulum. *Mol Microbiol,* **102,** 775-791.

Kaji, T., Kakui, K., Miyazaki, N., Murata, K. & Palmer, A. R. (2016) Mesoscale morphology at nanoscale resolution: serial block-face scanning electron microscopy reveals fine 3D detail of a novel silk spinneret system in a tube-building tanaid crustacean. *Front Zool,* **13,** 14.

Kleesattel, D., Crish, S. D. & Inman, D. M. (2015) Decreased Energy Capacity and Increased Autophagic Activity in Optic Nerve Axons With Defective Anterograde Transport. *Invest Ophthalmol Vis Sci,* **56,** 8215-8227.

Lewis, P. N., White, T. L., Young, R. D., Bell, J. S., Winlove, C. P. & Meek, K. M. (2016) Three-dimensional arrangement of elastic fibers in the human corneal stroma. *Exp Eye Res,* **146,** 43-53.

Martin, E. A., Woodruff, D., Rawson, R. L. & Williams, M. E. (2017) Examining Hippocampal Mossy Fiber Synapses by 3D Electron Microscopy in Wildtype and Kirrel3 Knockout Mice. *eNeuro,* **4**.

Meyer, K., Ostrenko, O., Bourantas, G., Morales-Navarrete, H., Porat-Shliom, N., Segovia-Miranda, F., Nonaka, H., Ghaemi, A., Verbavatz, J. M., Brusch, L., Sbalzarini, I., Kalaidzidis, Y., Weigert, R. & Zerial, M. (2017) A Predictive 3D Multi-Scale Model of Biliary Fluid Dynamics in the Liver Lobule. *Cell Syst*.

Mourik, M. J., Faas, F. G., Zimmermann, H., Eikenboom, J. & Koster, A. J. (2015) Towards the imaging of Weibel-Palade body biogenesis by serial block face-scanning electron microscopy. *J Microsc,* **259,** 97-104.

Mukherjee, K., Clark, H. R., Chavan, V., Benson, E. K., Kidd, G. J. & Srivastava, S. (2016) Analysis of Brain Mitochondria Using Serial Block-Face Scanning Electron Microscopy. *J Vis Exp*.

Neves, R. C. & Reichert, H. (2015) Microanatomy and development of the dwarf male of Symbion pandora (Phylum Cycliophora): new insights from ultrastructural investigation based on serial section electron microscopy. *PLoS One,* **10,** e0122364.

Nguyen, H. B., Thai, T. Q., Saitoh, S., Wu, B., Saitoh, Y., Shimo, S., Fujitani, H., Otobe, H. & Ohno, N. (2016) Conductive resins improve charging and resolution of acquired images in electron microscopic volume imaging. *Sci Rep,* **6,** 23721.

Nixon, F. M., Honnor, T. R., Clarke, N. I., Starling, G. P., Beckett, A. J., Johansen, A. M., Brettschneider, J. A., Prior, I. A. & Royle, S. J. (2017) Microtubule organization within mitotic spindles revealed by serial block face scanning electron microscopy and image analysis. *J Cell Sci,* **130,** 1845-1855.

Nkwe, D. O., Pelchen-Matthews, A., Burden, J. J., Collinson, L. M. & Marsh, M. (2016) The intracellular plasma membrane-connected compartment in the assembly of HIV-1 in human macrophages. *BMC Biol,* **14,** 50.

Ochs, M., Knudsen, L., Hegermann, J., Wrede, C., Grothausmann, R. & Muhlfeld, C. (2016) Using electron microscopes to look into the lung. *Histochem Cell Biol,* **146,** 695-707.

Ou, H. D., Deerinck, T. J., Bushong, E., Ellisman, M. H. & O'Shea, C. C. (2015) Visualizing viral protein structures in cells using genetic probes for correlated light and electron microscopy. *Methods,* **90,** 39-48.

Palaiologou, E., Goggin, P., Chatelet, D. S., Lofthouse, E. M., Torrens, C., Sengers, B. G., Cleal, J. K., Page, A. & Lewis, R. M. (2017) Serial block-face scanning electron microscopy of erythrocytes protruding through the human placental syncytiotrophoblast. *J Anat*.

Pfeifer, C. R., Shomorony, A., Aronova, M. A., Zhang, G., Cai, T., Xu, H., Notkins, A. L. & Leapman, R. D. (2015) Quantitative analysis of mouse pancreatic islet architecture by serial block-face SEM. *J Struct Biol,* **189,** 44-52.

Pinali, C., Bennett, H. J., Davenport, J. B., Caldwell, J. L., Starborg, T., Trafford, A. W. & Kitmitto, A. (2015) Three-dimensional structure of the intercalated disc reveals plicate domain and gap junction remodeling in heart failure. *Biophys J,* **108,** 498-507.

Pinali, C., Malik, N., Davenport, J. B., Allan, L. J., Murfitt, L., Iqbal, M. M., Boyett, M. R., Wright, E. J., Walker, R., Zhang, Y., Dobryznski, H., Holt, C. M. & Kitmitto, A. (2017) Post-Myocardial Infarction T-tubules Form Enlarged Branched Structures With Dysregulation of Junctophilin-2 and Bridging Integrator 1 (BIN-1). *J Am Heart Assoc,* **6**.

Pipkin, J. E., Bushong, E. A., Ellisman, M. H. & Kristan, W. B., Jr. (2016) Patterns and distribution of presynaptic and postsynaptic elements within serial electron microscopic reconstructions of neuronal arbors from the medicinal leech Hirudo verbana. *J Comp Neurol,* **524,** 3677-3695.

Plachno, B. J., Swiatek, P., Jobson, R. W., Malota, K. & Brutkowski, W. (2017) Serial block face SEM visualization of unusual plant nuclear tubular extensions in a carnivorous plant (Utricularia, Lentibulariaceae). *Ann Bot*.

Randles, M. J., Collinson, S., Starborg, T., Mironov, A., Krendel, M., Konigshausen, E., Sellin, L., Roberts, I. S., Kadler, K. E., Miner, J. H. & Lennon, R. (2016) Three-dimensional electron microscopy reveals the evolution of glomerular barrier injury. *Sci Rep,* **6,** 35068.

Rind, F. C., Wernitznig, S., Polt, P., Zankel, A., Gutl, D., Sztarker, J. & Leitinger, G. (2016) Two identified looming detectors in the locust: ubiquitous lateral connections among their inputs contribute to selective responses to looming objects. *Sci Rep,* **6,** 35525.

Sai, K., Wang, S., Kaito, A., Fujiwara, T., Maruo, T., Itoh, Y., Miyata, M., Sakakibara, S., Miyazaki, N., Murata, K., Yamaguchi, Y., Haruta, T., Nishioka, H., Motojima, Y., Komura, M., Kimura, K., Mandai, K., Takai, Y. & Mizoguchi, A. (2017) Multiple roles of afadin in the ultrastructural morphogenesis of mouse hippocampal mossy fiber synapses. *J Comp Neurol,* **525,** 2719-2734.

Sakaguchi, M., Miyazaki, N., Fujioka, H., Kaneko, O. & Murata, K. (2016) Three-dimensional analysis of morphological changes in the malaria parasite infected red blood cell by serial block-face scanning electron microscopy. *J Struct Biol,* **193,** 162-171.

Shami, G. J., Cheng, D., Huynh, M., Vreuls, C., Wisse, E. & Braet, F. (2016) 3-D EM exploration of the hepatic microarchitecture - lessons learned from large-volume in situ serial sectioning. *Sci Rep,* **6,** 36744.

Shomorony, A., Pfeifer, C. R., Aronova, M. A., Zhang, G., Cai, T., Xu, H., Notkins, A. L. & Leapman, R. D. (2015) Combining quantitative 2D and 3D image analysis in the serial block face SEM: application to secretory organelles of pancreatic islet cells. *J Microsc,* **259,** 155-164.

Smith, M. A., Xia, C. Z., Dengler-Crish, C. M., Fening, K. M., Inman, D. M., Schofield, B. R. & Crish, S. D. (2016) Persistence of intact retinal ganglion cell terminals after axonal transport loss in the DBA/2J mouse model of glaucoma. *J Comp Neurol,* **524,** 3503-3517.

Stucki, D. M., Ruegsegger, C., Steiner, S., Radecke, J., Murphy, M. P., Zuber, B. & Saxena, S. (2016) Mitochondrial impairments contribute to Spinocerebellar ataxia type 1 progression and can be ameliorated by the mitochondria-targeted antioxidant MitoQ. *Free Radic Biol Med,* **97,** 427-440.

Svensson, R. B., Herchenhan, A., Starborg, T., Larsen, M., Kadler, K. E., Qvortrup, K. & Magnusson, S. P. (2017) Evidence of structurally continuous collagen fibrils in tendons. *Acta Biomater,* **50,** 293-301.

Szczesny, S. E., Fetchko, K. L., Dodge, G. R. & Elliott, D. M. (2017) Evidence that interfibrillar load transfer in tendon is supported by small diameter fibrils and not extrafibrillar tissue components. *J Orthop Res*.

Thai, T. Q., Nguyen, H. B., Saitoh, S., Wu, B., Saitoh, Y., Shimo, S., Elewa, Y. H., Ichii, O., Kon, Y., Takaki, T., Joh, K. & Ohno, N. (2016) Rapid specimen preparation to improve the throughput of electron microscopic volume imaging for three-dimensional analyses of subcellular ultrastructures with serial block-face scanning electron microscopy. *Med Mol Morphol,* **49,** 154-162.

Vincent, A. E., Ng, Y. S., White, K., Davey, T., Mannella, C., Falkous, G., Feeney, C., Schaefer, A. M., McFarland, R., Gorman, G. S., Taylor, R. W., Turnbull, D. M. & Picard, M. (2016) The Spectrum of Mitochondrial Ultrastructural Defects in Mitochondrial Myopathy. *Sci Rep,* **6,** 30610.

Walker, A. S., Neves, G., Grillo, F., Jackson, R. E., Rigby, M., O'Donnell, C., Lowe, A. S., Vizcay-Barrena, G., Fleck, R. A. & Burrone, J. (2017) Distance-dependent gradient in NMDAR-driven spine calcium signals along tapering dendrites. *Proc Natl Acad Sci U S A,* **114,** E1986-E1995.

Wanner, A. A., Genoud, C. & Friedrich, R. W. (2016) 3-dimensional electron microscopic imaging of the zebrafish olfactory bulb and dense reconstruction of neurons. *Sci Data,* **3,** 160100.

Wernitznig, S., Rind, F. C., Polt, P., Zankel, A., Pritz, E., Kolb, D., Bock, E. & Leitinger, G. (2015) Synaptic connections of first-stage visual neurons in the locust Schistocerca gregaria extend evolution of tetrad synapses back 200 million years. *J Comp Neurol,* **523,** 298-312.

Wernitznig, S., Sele, M., Urschler, M., Zankel, A., Polt, P., Rind, F. C. & Leitinger, G. (2016) Optimizing the 3D-reconstruction technique for serial block-face scanning electron microscopy. *J Neurosci Methods,* **264,** 16-24.

White, T. L., Lewis, P., Hayes, S., Fergusson, J., Bell, J., Farinha, L., White, N. S., Pereira, L. V. & Meek, K. M. (2017a) The Structural Role of Elastic Fibers in the Cornea Investigated Using a Mouse Model for Marfan Syndrome. *Invest Ophthalmol Vis Sci,* **58,** 2106-2116.

White, T. L., Lewis, P. N., Young, R. D., Kitazawa, K., Inatomi, T., Kinoshita, S. & Meek, K. M. (2017b) Elastic microfibril distribution in the cornea: Differences between normal and keratoconic stroma. *Exp Eye Res,* **159,** 40-48.

Yamada, K., Young, R. D., Lewis, P. N., Shinomiya, K., Meek, K. M., Kinoshita, S., Caterson, B. & Quantock, A. J. (2015) Mesenchymal-epithelial cell interactions and proteoglycan matrix composition in the presumptive stem cell niche of the rabbit corneal limbus. *Mol Vis,* **21,** 1328-1339.
